# Immunogenicity of *Pasteurella multocida* and *Mannheimia haemolytica* outer membrane vesicles

**DOI:** 10.1016/j.ijmm.2013.05.001

**Published:** 2013-07

**Authors:** Sandro Roier, Judith C. Fenninger, Deborah R. Leitner, Gerald N. Rechberger, Joachim Reidl, Stefan Schild

**Affiliations:** Institute of Molecular Biosciences, University of Graz, Humboldtstraße 50, A-8010 Graz, Austria

**Keywords:** OMV, Intranasal, Fowl cholera, Pasteurellosis, Mouse model, Protection

## Abstract

*Pasteurella multocida* is able to cause disease in humans and in a wide range of animal hosts, including fowl cholera in birds, atrophic rhinitis in pigs, and snuffles in rabbits. Together with *Mannheimia haemolytica*, *P. multocida* also represents a major bacterial causative agent of bovine respiratory disease (BRD), which is one of the most important causes for economic losses for the cattle backgrounding and feedlot industry. Commercially available vaccines only partially prevent infections caused by *P. multocida* and *M. haemolytica*. Thus, this study characterized the immunogenicity of *P. multocida* and *M. haemolytica* outer membrane vesicles (OMVs) upon intranasal immunization of BALB/c mice. Enzyme-linked immunosorbent assays (ELISA) revealed that OMVs derived from *P. multocida* or *M. haemolytica* are able to induce robust humoral and mucosal immune responses against the respective donor strain. In addition, also significant cross-immunogenic potential was observed for both OMV types. Colonization studies showed that a potential protective immune response against *P. multocida* is not only achieved by immunization with *P. multocida* OMVs, but also by immunization with OMVs derived from *M. haemolytica*. Immunoblot and immunoprecipitation analyses demonstrated that *M. haemolytica* OMVs induce a more complex immune response compared to *P. multocida* OMVs. The outer membrane proteins OmpA, OmpH, and P6 were identified as the three major immunogenic proteins of *P. multocida* OMVs. Amongst others, the serotype 1-specific antigen, an uncharacterized outer membrane protein, as well as the outer membrane proteins P2 and OmpA were found to be the most important antigens of *M. haemolytica* OMVs. These findings are useful for the future development of broad-spectrum OMV based vaccines against BRD and other infections caused by *P. multocida* or *M. haemolytica*.

## Introduction

Bovine respiratory disease (BRD) is one of the most important causes for economic losses for the cattle backgrounding and feedlot industry due to elevated mortality rates, intensive costs for treatment and prevention as well as reduction of the carcass value (Confer, 2009, Fulton, 2009, McVey, 2009). The losses of the North American cattle industry alone are estimated to reach 1 billion US dollar per year (McVey, 2009, Miles, 2009, Whiteley et al., 1992).

Two members of the *Pasteurellaceae* family, *Mannheimia haemolytica* (formerly *Pasteurella haemolytica* biotype A) and *Pasteurella multocida*, represent major bacterial causative agents of respiratory disease in cattle, which are frequently associated with BRD (Confer, 2009, Griffin et al., 2010, Rice et al., 2007). In general, these bacteria are commensals in the nasopharynx of many domestic and wild animals including cats, dogs, horses, birds and cattle. The epidemiology of BRD is far from being understood and seems to be a complex and multi-factorial syndrome. The current model suggests that predisposing stress factors are necessary to induce the disease in otherwise asymptomatic carriers (Confer, 2009, Griffin, 2010, Griffin et al., 2010, Taylor et al., 2010). A non-exhaustive list of proposed stress factors includes transportation, weather changes, cold climate, wetness, dehydration, weaning, overcrowding or viral infections (Irwin et al., 1979, Lillie, 1974, Taylor et al., 2010). If colonized animals encounter such “stressors”, pathogens associated with BRD (e.g. *M. haemolytica* and *P. multocida*) can overwhelm the immune system of the host, rapidly proliferate in the nasopharynx, spread to the lower respiratory tract and establish severe lung infections. *P. multocida* has a relatively wide host range and can cause disease in a variety of animals besides cattle, for example atrophic rhinitis in pigs, snuffels in rabbits and fowl cholera in birds (Boyce and Adler, 2006, Harper et al., 2006). Especially fowl cholera outbreaks are a serious threat for poultry farms and wildlife birds (Bundesministerium für Gesundheit, 2008, Descamps et al., 2012, Leotta et al., 2006, Pedersen et al., 2003, Wang et al., 2009, Woo and Kim, 2006, Zhang et al., 2004). *M. haemolytica* has also been associated with severe pleuropneumonias in sheep and goats (Zecchinon et al., 2005). Although these bacteria are primarily considered as animal pathogens, wound infections in humans can occur upon contact with saliva of colonized animals, for example as a result from bites and scratches. Respiratory tract and invasive infections in humans are less common, but have been described for persons with close contact to animals and can cause severe complications in infants and immunocompromised patients (Henriksen and Jyssum, 1960, Kristinsson and Adam, 2007, Punpanich and Srijuntongsiri, 2012, Yaneza et al., 1991).

Vaccines to prevent animal infections caused by *M. haemolytica* and *P. multocida* are commercially available, but not all vaccines have consistently shown benefits in feedlot programs (Fulton, 2009, Griffin et al., 2010, Rice et al., 2007). The broad use of immunization as a preventive approach against BRD is also hampered by the economical burden for the farmers and cattle industry. Thus, ideally a BRD vaccine provides not only a long-lasting, protective immune response, but should also be cheap in production and administered without the presence of trained veterinarians. Recently, we have successfully characterized new vaccine candidates against the human pathogens *Vibrio cholerae* and nontypeable *Haemophilus influenzae* based on outer membrane vesicles (OMVs) (Bishop et al., 2010, Roier et al., 2012, Schild et al., 2009, Schild et al., 2008). OMVs are small spherical structures (approximately 10–300 nm), which are naturally released from the outer membrane (OM) of Gram-negative bacteria and can be purified from the culture supernatant by filtration and centrifugation steps (Ellis and Kuehn, 2010, Kulp and Kuehn, 2010, Mashburn-Warren and Whiteley, 2006). They can be seen as non-living facsimiles of the donor cell and therefore naturally contain important surface antigens as well as adjuvants including outer membrane proteins, periplasmic proteins, phospholipids, and the lipopolysaccharide (LPS). Not surprisingly, the immunogenic and protective properties of OMVs have now been tested and proven for several Gram-negative human pathogens, e.g. *V. cholerae*, nontypeable *H. influenzae*, *Neisseria meningitidis*, *Salmonella typhimurium*, *Borrelia burgdorferi*, *Bordetella pertussis*, and *Porphyromonas gingivalis* (Alaniz et al., 2007, Holst et al., 2009, Kesavalu et al., 1992, Roberts et al., 2008, Roier et al., 2012, Schild et al., 2008, Whitmire and Garon, 1993).

In the present study, we extended our research on OMVs as vaccine candidates to animal pathogens and investigated the induced immune responses upon intranasal immunization with OMVs derived from *P. multocida* using the murine model. *M. haemolytica* OMVs, which have been recently demonstrated to induce a protective immune response upon subcutaneous immunization in cattle (Ayalew et al., 2013) were tested in parallel to allow a direct comparison of the immunogenicity of OMVs from these two important animal pathogens. The humoral and mucosal immune responses were characterized by ELISA and immunoblot analysis. In addition we determined the most immunogenic antigens by immunoprecipitation and mass spectrometry. Finally, in case of *P. multocida* the protective immune response was evaluated by colonization studies with vaccinated and nonvaccinated control mice.

## Materials and methods

### Ethics statement

Female BALB/c mice (Charles River Laboratories) were used for all immunization experiments in strict accordance with the recommendations in the Guide for the Care and Use of Laboratory Animals of the National Institutes of Health, the national “Bundesgesetzblatt für die Republik Österreich”. The corresponding animal protocol (39/53/00 ex 2012/13) has been approved by the Austrian Federal Ministry of Science and Research Ref. II/10b and the Committee on the Ethics of Animal Experiments of the University of Graz. Mice were housed with food and water ad libitum and monitored under the care of full-time staff and in accordance with the rules of the Institute of Molecular Biosciences at the University of Graz. All animals were acclimated for 1 week before any procedures were carried out and were 9–11 weeks old at the start of the immunization.

### Bacterial strains and growth conditions

*P. multocida* P4881 was obtained from Lothar H. Wieler, Free University of Berlin, Berlin, Germany. The *M. haemolytica* strain SH789 (also designated as PHL213 or ATCC BAA-410) was kindly provided by Sarah K. Highlander, Baylor College of Medicine, Houston, USA. *P. multocida* P4881 was isolated from a case of bovine pneumonia (Rimler, 1996) and has been identified as a capsular serotype A strain (Ewers et al., 2006). *M. haemolytica* SH789 was isolated from the lung of a calf with bovine respiratory disease and represents a capsular serotype A1 strain (Fedorova and Highlander, 1997, Gioia et al., 2006). The spontaneous streptomycin-resistant (Sm^r^) derivative P4881-R of *P. multocida* P4881 as well as the natural Sm^r^ *M. haemolytica* SH789 were used in all experiments to allow the positive selection throughout the study including the challenge experiment. P4881-R was generated by plating an overnight culture of P4881 on brain heart infusion (BHI) agar supplemented with streptomycin. After 48 h, a single Sm^r^ colony was recovered, purified, and compared with the wild-type strain P4881 for their OM and OMV protein profiles as well as for their growth kinetics. No obvious differences were observed (data not shown).

Bacteria were grown at 37 °C with aeration on BHI agar supplemented with NAD and hemin-solution (stock-solution containing a mixture of hemin, l-histidine, and triethanolamine) or in BHI broth without NAD and hemin supplementation. When appropriate, streptomycin was added. Supplements were used in the following final concentrations: NAD, 10 μg/ml; hemin, 20 μg/ml; l-histidine, 20 μg/ml; triethanolamine, 0.08%; and streptomycin, 100 μg/ml.

### Preparation of outer membrane proteins (OMPs) and whole-cell lysates (WCL)

OMPs and WCL were prepared as previously published (Roier et al., 2012), with the exception that the cells were disrupted by homogenization with 0.1 mm glass beads in combination with a PowerLyzer™ 24 (MO BIO Laboratories, Inc.), applying three 1 min cycles at 3400 rpm with 1 min intervals on ice between each cycle. The protein concentrations of OMP and WCL preparations were determined by photometric measurements of the absorbances at 260 nm and 280 nm using a Beckman Coulter DU730 spectrophotometer in combination with a TrayCell (Hellma) and the Warburg–Christian equation.

### Preparation of OMVs

*P. multocida* and *M. haemolytica* OMVs were isolated as previously described for nontypeable *H. influenzae* OMVs (Roier et al., 2012). The protein concentrations of the OMV preparations were determined as mentioned above and adjusted to 2.5 μg/μl using PBS (pH 7.4).

### Immunization protocol

For the immunization with OMVs derived from *P. multocida* and *M. haemolytica*, mice were divided into two immunization groups. The first group received immunization mixture Pm-OMV consisting of OMVs derived solely from *P. multocida* P4881-R, whereas the second group was immunized with immunization mixture Mh-OMV consisting of OMVs derived solely from *M. haemolytica* SH789. Additionally, a nonvaccinated control group of mice sham immunized with PBS-treatment (pH 7.4) was housed in parallel with vaccinated mice for the duration of the experiment. Mice were intranasally immunized (5 μl per nostril) at days 0, 14, and 28 using a total of 25 μg OMVs (protein equivalent) in 10 μl PBS (pH 7.4). This intranasal immunization dose was based on previously published immunization studies using OMVs derived from nontypeable *H. influenzae*, *V. cholerae* and *N. meningitidis* (Dalseg et al., 1999, Leitner et al., 2013, Roier et al., 2012, Saunders et al., 1999, Schild et al., 2009, Schild et al., 2008). Mice were briefly anesthetized by inhalation of 2.5% isoflurane gas prior to all immunizations. No adjuvant was used for the entire study. To avoid potential effects by coprophagia between the different groups, mice of each immunization group as well as the nonvaccinated control mice were kept in separated cages. None of the animals died throughout the immunization studies and no significant differences in consumption of food and water were detected between cages harboring vaccinated and nonvaccinated control mice. Overall, three time-independent immunization rounds, with at least two mice per group, were performed. No differences in the induced immune responses or protection of the respective immunization groups could be detected for the independent immunization rounds.

### Collection and preparation of blood and stool samples

Blood samples were collected by lateral tail vein nick at day 0, 14, and 28, as well as by cardiac puncture at day 39. Additionally, three to five freshly voided fecal pellets per mouse were collected at day 39. Samples were processed as described previously (Schild et al., 2009, Schild et al., 2008). Serum samples and extracted Igs from the fecal pellets were stored at −70 °C.

### Challenge with *P. multocida*

All vaccinated and nonvaccinated control mice were intranasally challenged with approximately 1.5 x 10^8^ CFU/mouse of *P. multocida* P4881-R at day 38 for 24 h. The challenge was performed according to a previously published method with slight modifications (Roier et al., 2012). To prepare the inoculum, *P. multocida* P4881-R was grown in BHI broth to an optical density at 600 nm (OD_600_) of 1.2. Cells were harvested by centrifugation (3200 x *g*, 6 min, RT), resuspended in PBS (pH 7.4), and concentrated to an OD_600_ of 110 (equivalent to approximately 1.5 x 10^11^ CFU/ml). Subsequently, 1:10 dilutions in PBS (pH 7.4) were prepared. The first 1:10 dilution was used as the inoculum (approximately 1.5 x 10^10^ CFU/ml). In parallel, appropriate dilutions were plated on BHI plates supplemented with streptomycin and incubated at 37 °C for one day to determine the CFU/ml of the inoculum by back-calculating to the original suspension. Prior to challenge, mice were briefly anesthetized by inhalation of 2.5% isoflurane gas. Then mice were intranasally inoculated with approximately 1.5 x 10^8^ CFU/mouse using 10 μl (5 μl per nostril) of the inoculum. After 24 h, the mice were sacrificed and the nasopharynx from each mouse was removed by dissection. The nasopharynx was mechanically homogenized in BHI broth with 15% glycerol and appropriate 1:10 dilutions were plated on BHI plates supplemented with streptomycin. After incubation at 37 °C for one day, the colonization rates in CFU/nasopharynx were determined by back-calculation to the original volume of the homogenized nasopharynges.

### Quantitation of antibodies

Temporal immune responses, half-maximum total Ig titers and mucosal immune responses to OMV derived from *P. multocida* and *M. haemolytica* were determined by indirect enzyme-linked immunosorbent assay (ELISA) using 96-well ELISA microplates from BrandTech Scientific, Inc. (BRAND*plates*^®^ immunoGrade™). Immunoglobulin A (IgA), IgG1, and IgM antibodies as well as half-maximum total Ig titers (IgA, IgG, and IgM) to OMVs were quantified essentially as described before (Roier et al., 2012).

### SDS-PAGE and immunoblot analysis

The protein content of the OM, WCL, and OMVs was analyzed by sodium dodecyl sulfate-polyacrylamide gel electrophoresis (SDS-PAGE) (Laemmli, 1970) in combination with 12% gels using the Prestained Protein Marker Broad Range (New England Biolabs) as a molecular mass standard. *P. multocida* samples were boiled in SDS gel loading buffer A (50 mM TRIS, 2% SDS, 0.5% bromophenol blue, 25% glycerol, 600 mM DTT, pH 6.8) for 10 min before SDS-PAGE, whereas *M. haemolytica* samples were boiled in SDS gel loading buffer B (54 mM NaH_2_PO_4_, 55 mM EDTA, 5.5% SDS, 0.1% bromophenol blue, 25% glycerol, 600 mM DTT, pH 7.2). Protein bands were visualized according to Kang et al. (2002).

Immunoblot analysis was performed as described previously (Schild et al., 2008). Chemiluminescence detection was performed by using the Immun-Star™ WesternC™ Kit (Bio-Rad Laboratories) and subsequent exposure in a ChemiDoc XRS system (Bio-Rad Laboratories) in combination with Quantity One software (Bio-Rad Laboratories).

### Immunoprecipitation

Immunoprecipitation was performed by using the Dynabeads^®^ Protein G Immunoprecipitation Kit (Invitrogen) according to the manufacturer's manual. To avoid mouse-specific variations, sera collected on day 39 from all mice immunized with Pm-OMV or Mh-OMV were pooled and 16 μl of the respective mixture was used for binding of the antibodies to the beads. 16 μl of pooled serum collected on day 39 from the nonvaccinated control mice served as a negative control. 500 μl of an OMP preparation (0.4 μg/μl) from *P. multocida* P4881-R or 100 μl of an OMP preparation (0.5 μg/μl) from *M. haemolytica* SH789 was used as antigen. Proteins in the immunoprecipitations were separated by SDS-PAGE and analyzed by mass spectrometry.

### Protein analysis by mass spectrometry

The respective protein bands from the immunoprecipitation samples were excised from the gel and tryptically digested according to the method by Shevchenko et al. (1996). Peptide extracts were dissolved in 0.1% formic acid and separated on a nano-HPLC system (Ultimate 3000™, Dionex, Amsterdam, Netherlands). 70 μl samples were injected and concentrated on the loading column (LC Packings C18 PepMap™, 5 μm particle size, 100 Å pore size, 300 μm ID x 1 mm) for 5 min using 0.1% formic acid as isocratic solvent at a flow rate of 20 μl/min. The column was then switched to the nanoflow circuit, and the sample was loaded on the nanocolumn (LC-Packings C18 PepMap™, 75 μm inner diameter x 150 mm) at a flow rate of 300 nl/min and separated using the following gradient: solvent A: water, 0.3% formic acid, solvent B: acetonitrile/water 80:20 (v/v), 0.3% formic acid; 0–5 min: 4% B, after 40 min 55% B, then for 5 min 90% B and 47 min reequilibration at 4% B. The sample was ionized in a Finnigan nano-ESI source equipped with NanoSpray tips (PicoTip™ Emitter, New Objective, Woburn, MA, USA) and analyzed in a Thermo-Finnigan LTQ linear ion trap mass spectrometer (Thermo, San Jose, CA, USA). The MS/MS data were analyzed with SpectrumMill Rev. 03.03.084 SR4 (Agilent, Darmstadt, GER) software using a *P. multocida* Pm70, a *M. haemolytica* PHL213 (SH789) or a non-redundant proteome database from NCBI (National Center for Biotechnology Information, Bethesda, MD, USA; http://www.ncbi.nlm.nih.gov), respectively.

### Statistical analysis

Data were analyzed using the Kruskal–Wallis test followed by post hoc Dunn's multiple comparisons. Differences were considered significant at *P* values of <0.05. For all statistical analyses, GraphPad Prism version 5.01 for Windows (GraphPad Software) was used.

## Results

### Induction of humoral and mucosal immune responses after immunization with *P. multocida* or *M. haemolytica* OMVs

**Fig. 1:**
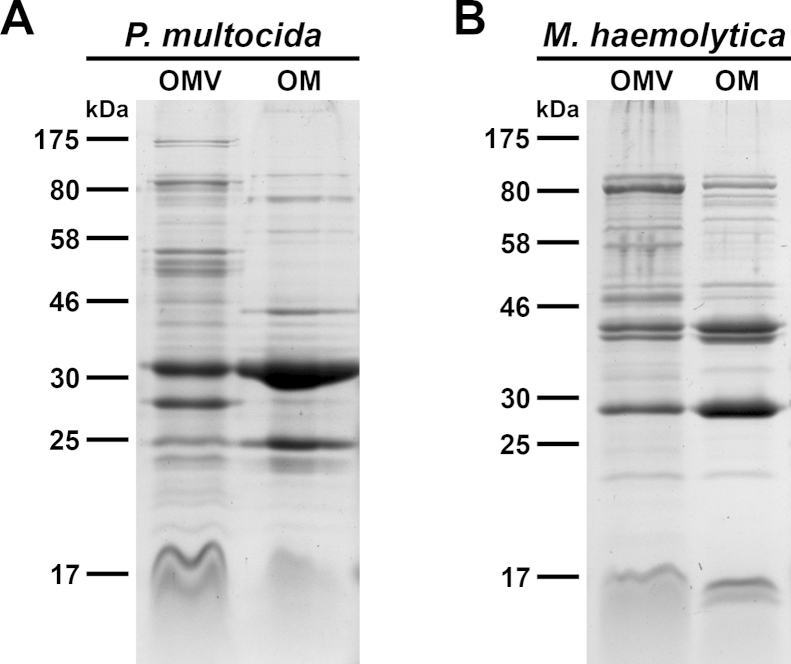
OMV and OMP profiles of *P. multocida* and *M. haemolytica*. The protein profiles of OMV and OM preparations from *P. multocida* (A) and *M. haemolytica* (B) were compared by SDS-PAGE followed by visualization of the protein bands according to Kang et al. (2002). Approximately 7 μg protein of each sample were loaded onto the gels. Lines to the left indicate the molecular masses of the protein standards in kDa.

The protein profiles of purified OMVs derived from *P. multocida* or *M. haemolytica* were compared with the respective OM preparations by SDS-PAGE in combination with Kang's staining method (Fig. 1). This analysis revealed similar profiles of the most abundant proteins between the respective OMV and OM preparations of *P. multocida* and *M. haemolytica*, indicating that these proteins of the OM are also present in the derived OMVs. Some protein bands were found to be over- or underrepresented in the OMV preparations compared to the OM preparations. This seems to be a characteristic feature of OMVs, which has also been described for another member of the *Pasteurellaceae* family (Roier et al., 2012) as well as for several other bacteria (Bauman and Kuehn, 2006, Haurat et al., 2011, Kato et al., 2002, Olofsson et al., 2010, Schild et al., 2008, Wensink and Witholt, 1981).

To investigate the immunogenicity of *P. multocida* OMVs in comparison to *M. haemolytica* OMVs, BALB/c mice were intranasally immunized with OMVs derived from *P. multocida* (Pm-OMV) or *M. haemolytica* (Mh-OMV) (see Materials and methods for details). PBS-treated mice served as a nonvaccinated control group. Immune responses were determined by ELISA using plates coated with either *P. multocida* or *M. haemolytica* OMVs. Thus, the immune response against the same species is provided by the Ig titers of the Pm-OMV group to *P. multocida* OMVs as well as the Mh-OMV group to the *M. haemolytica* OMVs. In addition, the cross-species immune response is provided by the Ig titers of the Pm-OMV group to *M. haemolytica* OMVs and vice versa.

**Fig. 2:**
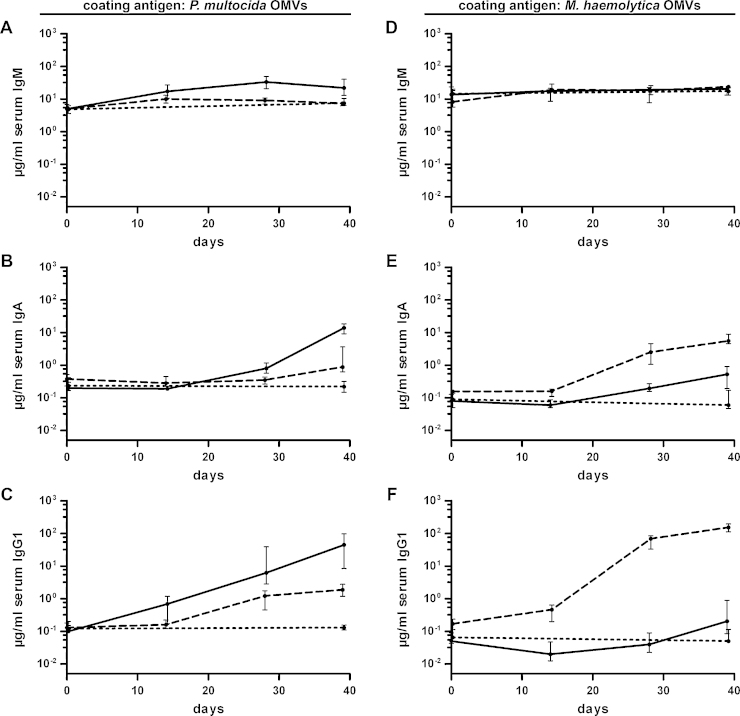
Temporal immune responses to OMVs derived from *P. multocida* and *M. haemolytica*. Shown are the median titers over time of IgM (A and D), IgA (B and D), and IgG1 (C and F) antibodies to *P. multocida* (A–C) and *M. haemolytica* OMVs (D–F). The temporal immune responses were determined by ELISA using sera from mice intranasally immunized with either Pm-OMV (solid line) or Mh-OMV (dashed line) as well as from nonvaccinated control mice (dotted line) (*n* ≥ 8 for each group). The error bars indicate the interquartile range of each data set for each time point.

Fig. 2 shows the temporal IgM, IgA, and IgG1 responses in sera to OMVs derived from *P. multocida* or *M. haemolytica*. The IgM, IgA, and IgG1 titers of the nonvaccinated control group were determined only for day 0 and 39, since no considerable differences were observed within this period. At day 0, the isotype-specific antibody titers to OMVs derived from *P. multocida* or *M. haemolytica* showed no significant differences between the three groups (*P* > 0.05; Kruskal–Wallis test and post hoc Dunn's multiple comparisons). Since IgM is the first Ig isotype expressed by mature B cells, an increase of the IgM titers is a first sign of an induction of a humoral immune response (Boes, 2000). Our group recently reported a pronounced induction of IgM titers of at least 10-fold after immunization with OMVs derived from nontypeable *H. influenzae* or *V. cholerae* (Roier et al., 2012, Schild et al., 2009, Schild et al., 2008). In the present study, the median IgM antibody titers to *P. multocida* OMVs showed only a mild increase over time and peaked at day 14 (Mh-OMV) or day 28 (Pm-OMV) followed by slight declines, most likely due to isotype switching (Fig. 2A). No temporal change in the IgM response was observed for both immunization groups when the ELISA plates were coated with *M. haemolytica* OMVs (Fig. 2D).

In contrast to IgM, the median IgA and IgG1 antibody titers to *P. multocida* (Fig. 2B and C) or *M. haemolytica* (Fig. 2E and F) of both immunization groups increased during the immunization period and showed the highest level at the endpoint of the experiment at day 39. Not surprisingly, the highest IgA and IgG1 titers to *P. multocida* OMVs at day 39 were detected for the Pm-OMV group, which were significantly higher compared to the nonvaccinated control as well as the Mh-OMV group (Fig. 2B and C; *P* < 0.05; Kruskal–Wallis test and post hoc Dunn's multiple comparisons). Accordingly, the Mh-OMV group exhibited the highest IgA and IgG1 titers to *M. haemolytica* OMVs at day 39, which were also significantly higher compared to the nonvaccinated control as well as the Pm-OMV group (Fig. 2E and F; *P* < 0.05; Kruskal–Wallis test and post hoc Dunn's multiple comparisons). In comparison to the respective nonvaccinated control group, the Mh-OMV immunization group exhibited a significant induction of IgA and IgG1 titers to *P. multocida* OMVs at day 39 and the Pm-OMV group showed significant higher IgA titers to *M. haemolytica* OMVs at day 39 (Fig. 2B, C and E; *P* < 0.05; Kruskal–Wallis test and post hoc Dunn's multiple comparisons). Thus, an induced IgA immune response across the two species could also be detected.

**Fig. 3:**
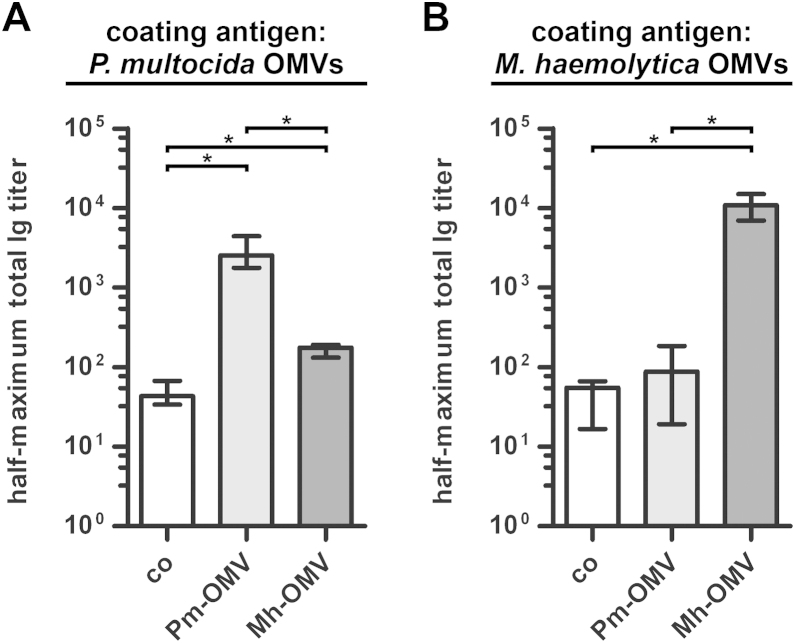
Half-maximum total Ig titers to OMVs derived from *P. multocida* and *M. haemolytica*. The median half-maximum total Ig titers to OMVs derived from *P. multocida* (A) and *M. haemolytica* (B) are shown. Sera collected at day 39 from mice intranasally immunized with either Pm-OMV or Mh-OMV as well as from nonvaccinated control mice (co) were analyzed by ELISA for quantitation of the half-maximum total Ig titers (*n* ≥ 8 for each group). The error bars indicate the interquartile range of each data set. Significant differences between the data sets are marked by asterisks (*P* < 0.05; Kruskal–Wallis test and post hoc Dunn's multiple comparisons).

To simultaneously detect IgM as well as all abundant subclasses of IgG and IgA, half-maximum total Ig titers in sera collected at day 39 from vaccinated and nonvaccinated control mice to OMVs derived from *P. multocida* (Fig. 3A) and *M. haemolytica* (Fig. 3B) were determined. ELISA plates coated with *P. multocida* OMVs revealed significantly different half-maximum total Ig titers between both immunization groups as well as the nonvaccinated control group (Fig. 3A; *P* < 0.05; Kruskal–Wallis test and post hoc Dunn's multiple comparisons). Concordant with the temporal immune response (Fig. 2B and C), the highest half-maximum total titers to *P. multocida* OMVs were detected in the Pm-OMV group, followed by the Mh-OMV group and the nonvaccinated control group. When the ELISA plates were coated with *M. haemolytica* OMVs, only the half-maximum total Ig titers of mice immunized with Mh-OMV, but not those of the Pm-OMV group, were significantly increased compared to the nonvaccinated control mice (Fig. 3B; *P* < 0.05; Kruskal–Wallis test and post hoc Dunn's multiple comparisons). This result is consistent with the trends observed for the IgG1 titers at day 39 (Fig. 2F), which are the most abundant Ig isotypes in the serum and therefore might have the most prominent impact on the half-maximum total Ig titers.

**Fig. 4:**
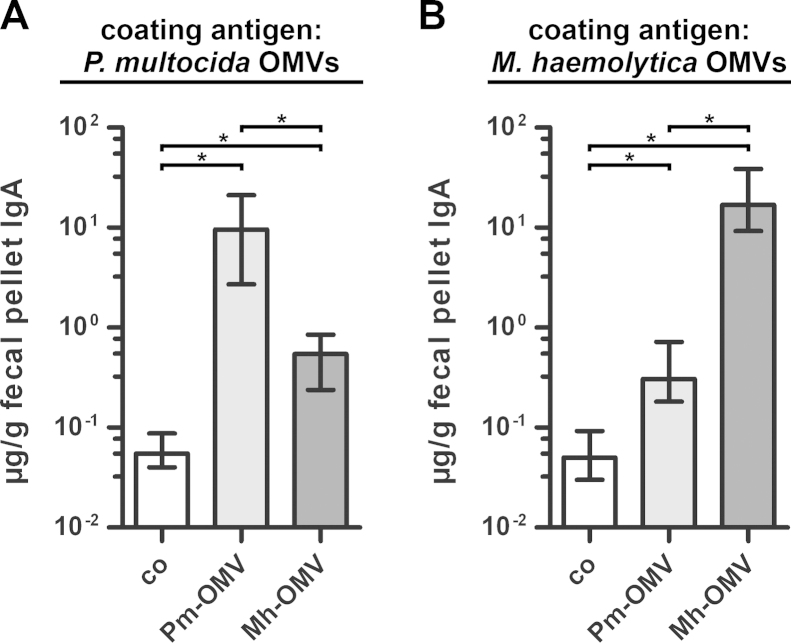
Mucosal immune responses to OMVs derived from *P. multocida* and *M. haemolytica*. Shown are the median IgA titers to OMVs derived from *P. multocida* (A) and *M. haemolytica* (B) extracted from fecal pellets collected at day 39. To determine the mucosal immune responses, fecal pellet extracts from mice intranasally immunized with either Pm-OMV or Mh-OMV as well as from nonvaccinated control mice (co) were analyzed by ELISA (*n* ≥ 8 for each group). The error bars indicate the interquartile range of each data set. Significant differences between the data sets are marked by asterisks (*P* < 0.05; Kruskal–Wallis test and post hoc Dunn's multiple comparisons).

Apart from the humoral immune responses, also the mucosal immune responses were analyzed by ELISA. In general, secretory antibodies to nasopharyngeal pathogens are often detected in body fluids that are obtained either by performing nasal washes using PBS or by collecting saliva after injection of pilocarpine to induce salivary secretion (Bertot et al., 2004, Hirano et al., 2003). However, all mice in this study were intranasally challenged, so that nasal washes or pilocarpine injections most likely would have interfered with nasopharyngeal colonization. Therefore, we used fecal pellets collected at day 39 to determine the mucosal immune responses, because it has been shown before that secretory IgA antibodies reflecting the mucosal immune response can also be found in feces and that IgA titers in feces correlate with those in saliva (Hirano et al., 2006, Schild et al., 2009, Vetvik et al., 1998). The secretory IgA titers from vaccinated and nonvaccinated control mice to OMVs derived from *P. multocida* and *M. haemolytica* are shown in Fig. 4A and B, respectively. Regardless of the OMV type coated with, both immunization groups showed significant increases in the secretory IgA titers compared to the nonvaccinated control group as well as significant differences between each other (Fig. 4; *P* < 0.05; Kruskal–Wallis test and post hoc Dunn's multiple comparisons). This trend of the mucosal immune response reflects the results obtained for the serum IgA titers at day 39 (Fig. 2B and E).

In summary, the highest titers were detected for the species-specific combination, represented by the Ig titer quantification of the Pm-OMV group to *P. multocida* OMVs or Mh-OMV group to *M. haemolytica* OMVs. Furthermore, a considerable induction of a cross-species immune response, especially for the humoral and secretory IgA titers could be observed.

### Specificity of the antibody response after immunization with *P. multocida* or *M. haemolytica* OMVs

**Fig. 5:**
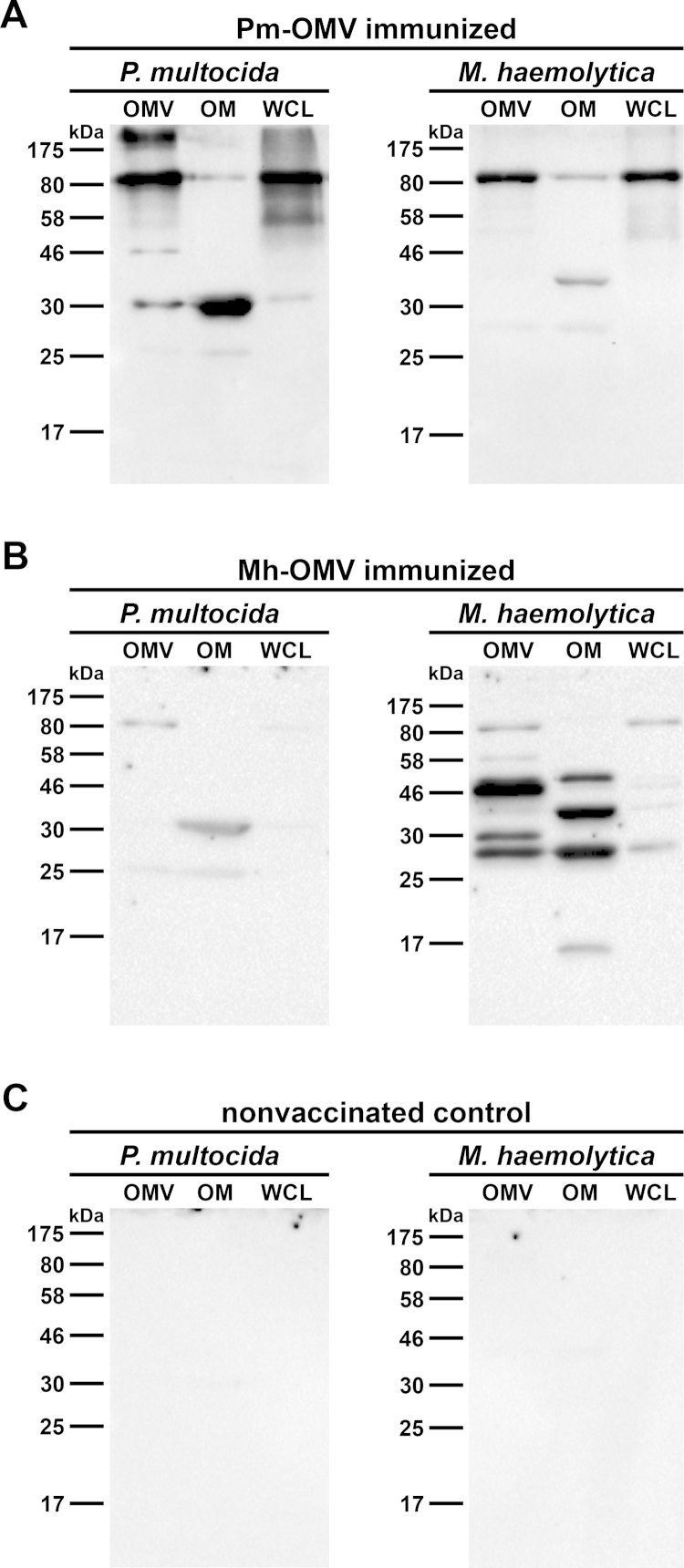
Immunoblot analysis of IgG reactivity in sera from vaccinated and nonvaccinated control mice. Representative immunoblots incubated with sera collected at day 39 from mice intranasally immunized with either Pm-OMV (A) or Mh-OMV (B) as well as from a nonvaccinated control mouse (C) are shown. Each immunoblot was loaded with OMV, OM, and WCL preparations (approx. 5 μg protein each) derived from either *P. multocida* or *M. haemolytica*. Lines to the left indicate the molecular masses of the protein standards in kDa.

Immunoblot analyses using OMV, OM, and WCL preparations derived from *P. multocida* and *M. haemolytica* as antigens were performed to test the specificity of the antibody response. The IgG reactivity was analyzed by incubating immunoblots with sera collected at day 39 from mice immunized with either Pm-OMV (Fig. 5A) or Mh-OMV (Fig. 5B) as well as from nonvaccinated control mice (Fig. 5C). No bands were detected on immunoblots using sera from nonvaccinated control mice (Fig. 5C). In contrast, immunoblots incubated with sera from Pm-OMV immunized mice (Fig. 5A) revealed up to four reactive bands with diverse intensities in the OMV, OM, and WCL protein profiles of *P. multocida.* In detail, a dominant reactive band of approx. 80 kDa was detected in the OMV and WCL sample, while another dominant reactive band located at approx. 30 kDa was detected in the OM sample. Accordingly, multiple bands were detected in the OMV, OM, and WCL protein profiles of *M. haemolytica* when the immunoblots were incubated with sera from Mh-OMV immunized mice (Fig. 5B). Here, three dominant reactive bands for the OMV and OM sample could be observed. While one band at approx. 28 kDa was detected in both samples, the other two dominant bands varied in size and were located at approx. 30 and 46 for the OMV sample as well as at approx. 35 and 50 kDa for the OM sample, respectively. Interestingly, also on immunoblots incubated with sera from Pm-OMV as well as from Mh-OMV immunized mice several faint bands were detected in the protein profiles of samples from *M. haemolytica* (Fig. 5A) and *P. multocida* (Fig. 5B), respectively. These faint bands essentially correlate with the dominant reactive bands in the OM protein profile of the respective strain, which were described above.

**Fig. 6:**
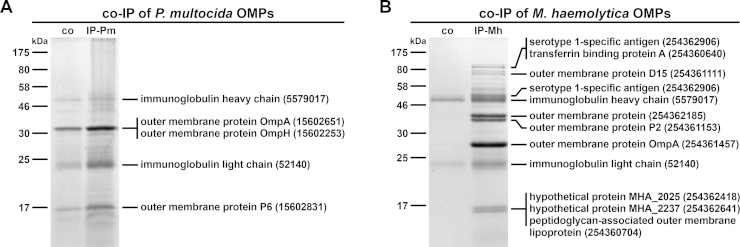
Immunoprecipitation using pooled sera from Pm-OMV or Mh-OMV immunized mice. Kang stained gels showing OMPs from *P. multocida* (A) or *M. haemolytica* (B) that co-immunoprecipitate (co-IP) with serum antibodies from nonvaccinated control mice (co) or from mice intranasally immunized with either Pm-OMV (IP-Pm) or Mh-OMV (IP-Mh) immobilized onto Dynabeads coupled with protein G. Therefore, pooled sera collected at day 39 from the respective vaccinated and nonvaccinated control mice were used. Proteins of the respective immunoprecipitation sample were identified by mass spectrometry and are indicated with their respective position on the gel, protein identities and accession numbers on the right. Lines to the left indicate the molecular masses of the protein standards in kDa.

In addition to immunoblot analyses, immunoprecipitation analyses were performed to identify the most immunogenic proteins of *P. multocida* and *M. haemolytica* OMVs. Therefore, pooled sera collected at day 39 from the respective vaccinated and nonvaccinated control mice were used as antibody sources and OM preparations of *P. multocida* or *M. haemolytica* served as target antigens. Fig. 6 shows SDS-PAGE profiles of OMPs from *P. multocida* (Fig. 6A) or *M. haemolytica* (Fig. 6B) that co-immunoprecipitate with serum antibodies from Pm-OMV (Fig. 6A; IP-Pm) as well as Mh-OMV (Fig. 6B; IP-Mh) immunized mice. In both cases, sera from nonvaccinated control mice were used as a negative control. The importance of the negative control is demonstrated by the faint bands appearing in both immunoprecipitations using sera from nonvaccinated control mice (Fig. 6A and B). These faint bands could be explained by intrinsic levels of natural IgM antibodies to *P. multocida* and *M. haemolytica*, which also bind to the protein G used for the immunoprecipitation. Their presence is indicated by the relatively high initial IgM titers of the vaccinated and nonvaccinated control mice (Fig. 2A and D). Additionally, it cannot be excluded that the OM preparations used in the immunoprecipitations contained protein complexes, which were not completely dissociated and could have been pulled down as aggregates. Therefore, always in comparison with the protein profiles obtained by immunoprecipitations using sera from nonvaccinated control mice, only the most intensive protein bands of the immunoprecipitations using sera from immunized mice were excised and subjected to mass spectrometry. These analyses revealed some immunogenic proteins of OMVs derived from *P. multocida* (Fig. 6A) and *M. haemolytica* (Fig. 6B), which are indicated with their respective position in the gels provided in Fig. 6. Due to the nature of the immunoprecipitation method, the mouse immunoglobulin heavy and light chains were always co-eluted with the antigens in all immunoprecipitations (Fig. 6). The positions of the immunogenic proteins also correlate with the locations of the dominant reactive bands in the OM protein profiles of *P. multocida* (Fig. 5A) and *M. haemolytica* (Fig. 5B) in the respective immunoblots. In detail, the outer membrane proteins OmpA, OmpH, and P6 were identified as immunogenic proteins of *P. multocida* OMVs. Noteworthy, OmpA and OmpH were found to have identical migration patterns in a SDS-PAGE and showed just one overlapping protein band at approx. 30 kDa (Fig. 6A). In comparison with OMVs derived from *P. multocida*, *M. haemolytica* OMVs seem to induce a more complex immune response, since at least nine OMPs were identified that co-immunoprecipitate with serum antibodies from Mh-OMV immunized mice (Fig. 6B). Amongst those important antigens, the serotype 1-specific antigen, an uncharacterized outer membrane protein, as well as the outer membrane proteins P2 and OmpA were found to be the most enriched proteins compared to the negative control. Concordant with the dominant reactive bands in the OM protein profile of *M. haemolytica* identified in immunoblots incubated with sera from Mh-OMV immunized mice (Fig. 5B), these immunogenic proteins were located at approx. 50, 35, and 28 kDa (Fig. 6B).

### Reduced colonization by *P. multocida* after immunization with *P. multocida* or *M. haemolytica* OMVs

**Fig. 7:**
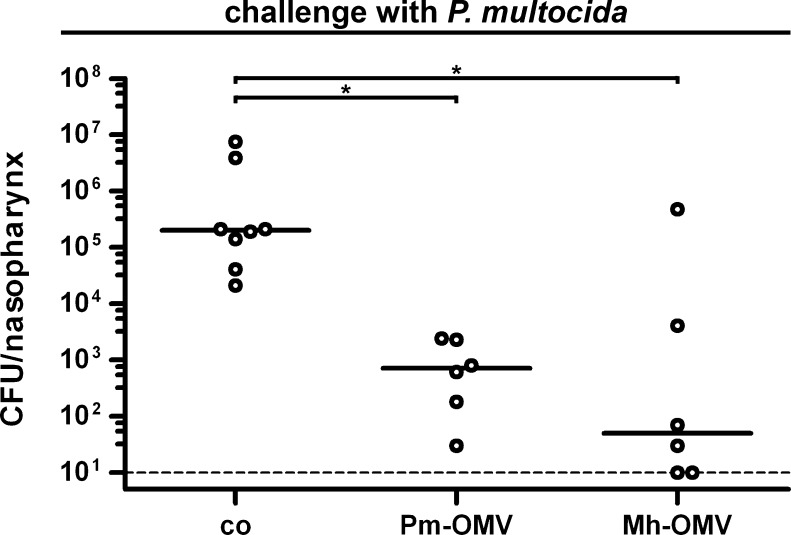
Challenge of vaccinated and nonvaccinated control mice with *P. multocida*. Shown are the nasopharyngeal colonization rates in recovered CFU per nasopharynx for mice intranasally immunized with either Pm-OMV or Mh-OMV as well as for nonvaccinated control mice (co). Mice were intranasally challenged with *P. multocida* for 24 h. Each circle represents the recovered CFU per nasopharynx from one mouse. The horizontal bars indicate the median of each data set. If no bacteria could be recovered, then the values were set to the limit of detection of 10 CFU/nasopharynx (indicated by the dashed line). Since independent immunization rounds were performed, the exact infection doses ranged from 1.0 x 10^8^ to 1.7 x 10^8^ CFU/mouse. Significant differences between the data sets are marked by asterisks (*P* < 0.05; Kruskal–Wallis test and post hoc Dunn's multiple comparisons).

Challenge experiments were performed to investigate the potential of the immunization mixtures Pm-OMV and Mh-OMV to reduce the nasopharyngeal colonization rates of *P. multocida* in mice upon challenge. Therefore, mice intranasally immunized with either Pm-OMV or Mh-OMV as well as nonvaccinated control mice were intranasally challenged with approximately 1.5 x 10^8^ CFU/mouse of *P. multocida* at day 38 for 24 h. This infection dose was based on preliminary experiments showing that a dose of about 10^8^ CFU/mouse ensures a stable colonization of *P. multocida* over 24 h (data not shown). Fig. 7 shows the nasopharyngeal colonization rates in recovered CFU per nasopharynx for vaccinated and nonvaccinated control mice after challenge with *P. multocida*. All nonvaccinated control mice were stably colonized with a median colonization rate of 2 x 10^5^ CFU/nasopharynx. In contrast, both immunization groups showed significant and at least 300-fold reductions in their median nasopharyngeal colonization rates compared to the nonvaccinated control group (*P* < 0.05; Kruskal–Wallis test and post hoc Dunn's multiple comparisons). Interestingly, no significant differences in the colonization rates between the Pm-OMV and the Mh-OMV group were observed, although the Mh-OMV group showed a higher variability in the colonization rates compared to the Pm-OMV group. In summary, this data indicates that the induced humoral and mucosal immune responses in mice immunized with either Pm-OMV or Mh-OMV are sufficient to significantly reduce the nasopharyngeal colonization rates of *P. multocida* after challenge.

## Discussion

The aim of this study was to investigate whether OMVs derived from animal pathogens are suitable vaccine candidates. It has been recently reported that subcutaneous immunization with *M. haemolytica* OMVs induces a protective immune response in calves (Ayalew et al., 2013). In the present study we focused on the non-invasive intranasal immunization route and included OMVs derived from *P. multocida*, which is another important pathogen associated with the BRD complex (Confer, 2009). In addition, *P. multocida* causes diseases in a variety of wild and domestic animals (Boyce and Adler, 2006) as well as in humans (Kristinsson and Adam, 2007). The secretion of OMVs by *P. multocida* and *M. haemolytica* has been described previously (Ayalew et al., 2013, Marandi and Mittal, 1995, Myhrvold et al., 1992). In the present study, we analyzed the OMV and OM protein profiles of *P. multocida* and *M. haemolytica* upon using a well-established method for OMV isolation (Roier et al., 2012). This analysis revealed similar protein profiles between the respective OMV and OM preparations of *P. multocida* and *M. haemolytica*, with some over- or underrepresented proteins in the OMV preparations compared to the OM preparations. This characteristic feature of OMVs has also been described for another member of the *Pasteurellaceae* family (Roier et al., 2012) as well as for several other bacteria (Bauman and Kuehn, 2006, Haurat et al., 2011, Kato et al., 2002, Olofsson et al., 2010, Schild et al., 2008, Wensink and Witholt, 1981).

In addition, we performed nasopharyngeal immunization and colonization studies with BALB/c mice to investigate the immunogenic and protective properties of *P. multocida* OMVs. Mice immunized with OMVs derived from *M. haemolytica* served as an internal control to evaluate and compare the induced immune responses between the groups immunized with either *P. multocida* OMVs (Pm-OMV) or *M. haemolytica* OMVs (Mh-OMV). We currently can only speculate for the reasons why the characteristic peak of IgM during the immunization is unincisive in the present study. It should be noted that the median IgM titers at day 0 of both immunization groups and the nonvaccinated control group were at least 20-fold increased compared to the respective median IgA and IgG1 titers. A similar tendency was observed previously in a nontypeable *H. influenzae* OMV vaccination study (Roier et al., 2012). As already suggested for nontypeable *H. influenzae*, we speculate that these BALB/c mice could have been in contact with or colonized with closely related bacterial species like other *Pasteurellaceae* family members. Thus, a certain intrinsic level of low-affinity, cross-reacting, natural IgM antibodies to bacteria of the *Pasteurella* and *Mannheimia* genera might already exists in naïve BALB/c mice and could mask a further stimulation upon immunization with OMVs.

Not surprisingly, the Pm-OMV and Mh-OMV immunization groups induced robust immune responses against the respective donor strain. In detail, ELISA plates coated with *P. multocida* OMVs showed that both immunization mixtures significantly increased the serum IgA, the serum IgG1, the half-maximum total Ig titers and the secretory IgA titers to *P. multocida* compared to the nonvaccinated control group. Consequently, both immunization groups showed significant reductions in their nasopharyngeal colonization rates after challenge with *P. multocida*. Although the Pm-OMV immunized mice induced significantly higher levels of humoral and mucosal immune responses to *P. multocida* compared to the Mh-OMV group, no significant differences in the colonization rates between both immunization groups upon challenge with *P. multocida* could be observed. This indicates that already relatively low levels of induced Ig titers as detected for the Mh-OMV group are sufficient to reduce the nasopharyngeal colonization rates of *P. multocida*, and that such high titers as observed for the Pm-OMV immunized group are not necessarily required. Taken together, these results demonstrate that a potential protective immune response against *P. multocida* is not only achieved by immunization with *P. multocida* OMVs, but also by immunization with OMVs derived from *M. haemolytica*.

Unfortunately, we were not able to ensure a stable nasopharyngeal colonization of *M. haemolytica* over 24 h in mice (data not shown). However, we quantified the induced humoral and mucosal immune responses to *M. haemolytica* after immunization with either Pm-OMV or Mh-OMV, by coating the ELISA plates with *M. haemolytica* OMVs. We were able to show that the Mh-OMV immunization mixture significantly increased the humoral and mucosal immune responses to *M. haemolytica* compared to the nonvaccinated control group. The level of the induced immune responses was comparable to that of the induced immune responses to *P. multocida* after immunization with Pm-OMV. Based on these results and the fact that *M. haemolytica* OMVs induce a protective immunity in calves (Ayalew et al., 2013), one could argue that an immunization with *M. haemolytica* OMVs would have also led to a protection against *M. haemolytica* in mice and that *P. multocida* OMVs have a high potential to stimulate a protective immunity against *P. multocida* in cattle. Additionally, the present study revealed that immunization with *P. multocida* OMVs significantly increases the serum IgA-titers as well as the secretory IgA titers to *M. haemolytica* OMVs compared to the nonvaccinated control group. Since a recent vaccination study using nontypeable *H. influenzae* OMVs has indicated that induced humoral IgA and especially mucosal IgA responses could be the most important antibody responses for protection against nontypeable *H. influenzae* infections (Roier et al., 2012), one could speculate that this might also be true for a potential protection against *M. haemolytica* after immunization with *P. multocida* OMVs. Future studies have to investigate, if the initial results and conclusions of the present study using the mouse model also hold true in other animal systems (e.g. cattle) and if a cross-protection against *P. multocida* and *M. haemolytica* can be achieved by using OMVs derived from just one species or if a mixture of *P. multocida* and *M. haemolytica* OMVs is needed.

In addition, the present study also analyzed the specificity of the induced antibody response and identified the most immunogenic proteins of *P. multocida* and *M. haemolytica* OMVs. Immunoblot analyses demonstrated that Pm-OMV immunized mice showed just one dominant reactive band in the OM protein profile of *P. multocida*, whereas Mh-OMV immunized mice revealed three dominant reactive bands in the OM protein profile of *M. haemolytica*. These results indicate that *M. haemolytica* OMVs contain more proteins that can serve as antigens compared to OMVs derived from *P. multocida* and thus induce a more complex immune response. Immunoprecipitation analyses confirmed this assumption, since at least nine different immunogenic proteins of *M. haemolytica* OMVs but only three important antigens of *P. multocida* OMVs could be elucidated. The outer membrane proteins OmpA, OmpH, and P6 were identified as the three major immunogenic proteins of *P. multocida* OMVs. Previous studies have shown that especially OmpA and OmpH are the major immunogenic proteins of *P. multocida* (Ataei et al., 2009, Gong et al., 2013, Vasfi Marandi and Mittal, 1997). It was also demonstrated previously that OmpA and OmpH show high and overlapping molecular mass heterogeneities in different *P. multocida* isolates ranging from 33 to 38 kDa (Davies et al., 2003, Davies et al., 2004). In the case of *P. multocida* strain P4881-R, which was used in the present study, OmpA and OmpH seem to have similar molecular masses, since they could not be separated due to different electrophoretic motility patterns. Therefore, the dominant reactive band in the OM protein profile of *P. multocida* identified in immunoblots incubated with sera from Pm-OMV immunized mice seems to be a result of the combined immunogenic potential of OmpA and OmpH.

Among the nine immunogenic proteins of *M. haemolytica* OMVs, five were found to be moderately enriched compared to the negative control in the immunoprecipitations using OMPs from *M. haemolytica*. These include the peptidoglycan-associated outer membrane lipoprotein, the hypothetical proteins MHA_2237 and MHA_2025, the outer membrane protein D15, and the transferrin binding protein A. Four highly immunogenic antigens of *M. haemolytica* OMVs were identified based on the most intense bands in the immunoprecipitation analyses, which correlate with the migration pattern of the dominant reactive bands in the OM protein profile of *M. haemolytica* identified in immunoblots incubated with sera from Mh-OMV immunized mice. These include the serotype 1-specific antigen, an uncharacterized outer membrane protein, as well as the outer membrane proteins P2 and OmpA. Especially OmpA and the serotype 1-specific antigen have previously been shown to be highly immunogenic in mice and cattle and are thus considered to be potential *M. haemolytica* vaccine candidates (Ayalew et al., 2011). Interestingly, OmpA from *M. haemolytica* and *P. multocida* share a sequence identity of 55%. Thus, mice immunized with *M. haemolytica* OMVs could have raised anti-OmpA antibodies that recognize also OmpA from *P. multocida*. This assumption is supported by the immunoblot results showing a faint band at approx. 30 kDa in the OM protein sample of *P. multocida*, when the immunoblots were incubated with sera from Mh-OMV immunized mice. The location of this faint band correlates with the *P. multocida* OmpA band identified by immunoprecipitation. This could explain the potential cross-protection against *P. multocida* in mice upon immunization with Mh-OMV. Furthermore, one could extend this idea and speculate that OmpA is also an important protective antigen of *P. multocida* OMVs. However, it cannot be excluded that additional antigens exist, which could also lead to the induction of a protective immunity against *P. multocida* in mice upon immunization with Pm-OMV. Most promising candidates would be OmpH and P6, which have also been identified by immunoprecipitation. Future studies are needed to address this question in more detail.

In conclusion, this study suggests that OMVs derived from *P. multocida* have a high potential to act as a vaccine against *P. multocida* infections like BRD in cattle or fowl cholera in avian hosts. Even without the use of a mucosal adjuvant, high titer humoral and mucosal immune responses to *P. multocida* and *M. haemolytica* OMVs have been shown. Probably OMVs provide a unique mixture of different surface antigens in their native conformation, which is a perfect blend for the induction of a robust immune response. In this context, the present study has elucidated several immunogenic proteins of OMVs derived from *P. multocida* or *M. haemolytica*. We used a non-invasive administration strategy by intranasal immunization, which could offer the opportunity for a vaccine application by inhalation in animal farms and consequently may not require trained health-care staff. Interestingly, isolated OMVs are quite temperature-stable and retain their immunogenic potential over months (Bishop et al., 2010, Schild et al., 2008). In summary, OMVs are stable, naturally released antigen delivery vehicles with a heterogeneous mixture of diverse components, which might be advantageous over vaccines solely based on purified recombinant proteins. Therefore, the results of the present study are useful for the future development of a broad-spectrum OMV based vaccine against BRD.
